# Depletion of proBNP_1-108_ in Patients with Heart Failure Prevents Cross-Reactivity with Natriuretic Peptides

**DOI:** 10.1371/journal.pone.0075174

**Published:** 2013-09-17

**Authors:** François Roubille, Delphine Delseny, Jean-Paul Cristol, Delphine Merle, Nicolas Salvetat, Catherine Larue, Jean-Marc Davy, Florence Leclercq, Jean-Luc Pasquie, Luc Guerrier, Jeannette Fareh, Anne-Marie Dupuy

**Affiliations:** 1 Cardiology Department, University Hospital of Montpellier, Université de Montpellier 1, Montpellier, France; 2 Research Center, Montreal Heart Institute, Université de Montréal, Montreal, Quebec, Canada; 3 Department of Biochemistry, University Hospital of Montpellier, Université de Montpellier 1, Montpellier, France; 4 CNRS UMR3145 Bio-Rad, SysDiag, Montpellier, France; 5 Bio-Rad Laboratories, Marnes la Coquette, France; Inserm, France

## Abstract

**Background:**

After synthesis by cardiomyocytes, precursor proBNP_1-108_ is cleaved into NT-proBNP and BNP. Recently, cross-reactivity between these assays was discussed. The aim of this study was to characterize the cross-reactivities, through a new biochemical innovative approach consisting in the total depletion of the circulating proBNP_1-108_ in patients with heart failure (HF).

**Methods:**

This prospective study included 180 patients with chronic HF. BNP and NT-proBNP were dosed with commercial kits. ProBNP_1-108_ was determined using an ELISA research assay specific to the precursor. ProBNP_1-108_ depletion was performed by immunocapture with a specific antibody targeting exclusively the ProBNP_1-108_ hinge region. ProBNP_1-108_, BNP and NT-proBNP levels were determined before and after depletion using this process in HF patients.

**Results:**

Mean age was 74.34 +/-12.5 y, and 69% of patients were males. NYHA classes II and III were the most frequent (32% and 45% respectively). Before depletion, ProBNP_1-108_, NT-proBNP and BNP levels were 316.8+/-265.9 pg/ml; 6,054.0+/-11,539 pg/ml and 684.3+/-82.1 pg/ml respectively, and were closely correlated with NHYA classes. After immuno-depletion, proBNP_1-108_ was decreased in mean by 96% (p<0.0001), BNP by 53% (p<0.0001) and NT-proBNP by 5%. The relationship between BNP or NT-proBNP and NHYA classes remained unchanged.

**Conclusion:**

Current BNP and NT-proBNP assays measured as well proBNP molecule. This cross reactivity percentage has been controversial. Thanks to the removal of circulating proBNP_1-108_ with our immunodepletion process, we are now able to assess the remaining “true” BNP and NT-proBNP molecules and further evaluate their clinical relevance.

## Introduction

The human BNP (B-type Natriuretic Peptide) gene encodes for a pre-proBNP molecule constituted of 134 amino acids. The pre-proBNP is cleaved out of the proBNP_1-108_. ProBNP_1-108_ was initially considered to be cleaved inside the cardiomyocytes into NT-proBNP (the biologically inactive NH2-terminal peptide fragment proBNP_1-76_) and the BNP (the biologically active hormone) [1,2]; both are more and more useful for clinical application [1,3,4], including diagnosis [3], treatment [3], prognosis [3], follow-up of patients with cardiac involvement, mainly heart failure (HF). However, intact proBNP_1-108_ was recently evidenced in plasma in significant amounts in patients with HF [2,5-7] with a lower biological activity than the BNP. In addition, cross-reactivity with the precursor intact proBNP_1-108_ between the commercial assays for BNP or NT-proBNP is high, with an inter-tests recovery ranging from 5 to 38% or 0-249% respectively [8], challenging the analytical specificities of available commercial kits, although at different levels. Recently, Nishikimi et al. described a direct immunochemiluminescent assay for proBNP and total BNP in 116 healthy subjects and in 32 patients with HF [9].

Little is known about such cross-reactivities in clinical settings, although this point appears critical to better characterize what routinely-used immunoassays measure, to better interpret results, and increase the clinical signification of the natriuretic peptides. In particular, the process we propose could help to better classify patients with atypical profiles and help in medical decision-making for appropriate therapy.

In this study, we aimed at characterizing the cross-reactivities of the natriuretic peptides assays, through a new biochemical approach consisting in the total immunodepletion of the circulating proBNP_1-108_ in patients at various stages of HF.

## Methods

Between May 2010 and February 2011, 180 patients with Heart Failure (HF) were prospectively included in a single University Hospital (CHRU Montpellier, France). The study was performed according to the Declaration of Helsinki (revised version of Somerset West, Republic of South Africa, 1996) and according to the European Guidelines of Good Clinical practice (version 11, July 1990) and French laws. The study protocol was approved by the local Ethics Committee of the University Hospital of Montpellier, written informed consent was obtained from all patients and the biological collection registered by the French government (research Ministery, # DC-2009-1052).

To be eligible to the study, the patients were previously (at least 6 months before the inclusion) diagnosed with acute or chronic HF, as recommended by the European Society of Cardiology [3]. Main inclusion criteria were the ability to give informed consent, age>18 years and confirmed diagnosis of HF, irrespectively of the cause or treatments. Main exclusion criteria were unstable angina or acute coronary syndrome in the past month, cardiac surgery and chemotherapy.

Venous blood was collected in EDTA tubes and was immediately centrifuged (the samples are transported a mean total delay of less than 3 hours (all inclusive until frozen); in the biochemistry lab 95% are treated in less than an hour and a half and 50% in less than one hour) and frozen (-80°C) until tested, three years later.

This was the first thawing.

The BNP levels were determined using an immunochemilumiscent method applied on the Access 2® analyzer (Beckman Coulter, Villepinte, France). Reagents, calibrators and controls were used according to the Biosite Package Insert. The NT-proBNP levels were determined using an inmmuno-electrochemiluminescence assay on the on the Cobas8000/e602® immunochemistry system (Roche Diagnostics, Meylan, France).

ProBNP was determined by an ELISA (Enzyme Linked ImmunoSorbent Assay) method for research use only as previously well described by Giuliani et al. [7] and Waldo et al. [10]. The limits of the detection as provided by the manufacturer are respectively 5 pg/ml for the NT-proBNP and 1 pg/ml for the BNP.

The proposed proBNP ELISA method is mainly characterized by the absence of cross-reactivity with circulating BNP and NT-proBNP, allowing specific determination of the proBNP_1-108_ form [7]. The proBNP_1-108_ removal was performed thanks to an immunodepletion system, consisting of NHS-activated beads (Affi-Gel 10, Bio-Rad Laboratories, hercules, CA, USA) coupled to a solution of proBNP-specific antibody (5 mg) according to manufacturer’s instructions. The mice monoclonal antibody binds specifically the proBNP hinge region or anti-irrelevant (myoglobin) antibody. After bead washing, EDTA samples were added to proBNP_1-108_ coated beads (5:10 ratio) for the depletion step (30 min at 4°C). After a rapid spin column centrifugation (500 rpm), the immunodepleted supernatant was collected for BNP, NTproBNP and proBNP_1-108_ measurements using the adapted platforms.

### Statistical analysis

All statistics and figures were performed with R 2.15.0 (www.R-project.org) or GraphPad Prism (version 4.0, GraphPad Software, San Diego California USA). Results are expressed as means with standard deviation except for study of reduction in peptides, presented as median and interquartile ranges, as more representative of the values’ dispersion. Comparisons were performed through Student t-tests or Wilcoxon rank-sum test depending on the normal distribution (Shapiro Wilks test) and homoscedasticity (F-test) of peptides, especially to analyze statistical significance of change-in-peptides comparisons. All data distributions are illustrated as medians and box plots for each peptide. Correlations were calculated with Spearman tests and Bland-Altman analysis to have the magnitude of difference in BNP and NTproBNP between before and after proBNP depletion. Distributions in different classes were compared using Kruskal-Wallis test.

## Results

### Mains Main characteristics of the population

Main characteristics of the population are described in the Table **1**. The mean age of the 180 subjects was 74.34 +/-12.5y, and a majority was males (69%). Conventional risk factors were first hypertension (61%) and overweight (57%), whereas tabagism (34%) and diabetes (29%) were less frequent. NYHA classes II and III were the most frequent (32% and 45% respectively). Ischemic cardiomyopathy was found in more than half of the patients. Treatments indicated in patients with HF followed ESC guidelines, with 83% of the patients under beta-blockers, and about ¾ under ACE-inhibitors, but only 24% under mineraloreceptor antagonists.

**Table 1:** Baseline characteristics of the study population.

**Characteristics**	**Mean+/-SD or n/total (%**)
**Main characteristics**	
Age (y)	74.3+/-12.5
Sex (Males)	125/180 (69)
Heart rate (bpm)	80.8+/-23.95
LVEF (%)	37.0+/-13.9
**Cardiovascular risk factors**	
Overweight	97/171 (57)
BMI^†^	26.8+/-0.4
Hypertension	87/143 (61)
Tabagism	49/143 (34)
Diabetes	52/143 (36)
Dyslipidemia	62/143 (43)
**NYHA**	
I	8/171 (5)
II	55/171 (32)
III	77/171 (45)
IV	31/171 (18)
**Etiology of the cardiopathy**	
Ischemic	103/180 (57)
Dilated cardiomyopathy	31/180 (17)
Valvulopathy	18/180 (10)
Rhythmic disease	16/180 (9)
Hypertrophic	7/180 (4)
Mixed disease	5/180 (3)
**Treatments**	
Beta-blockers	135/163 (83)
ACE-inhibitors/ARB	126/163 (77)
Loop-diuretics	120/164 (73)
Eplerenone-spironolactone	39/163 (24)

† The body-mass index is the weight in kilograms divided by the square of the height in meters.

SD: standard deviation, LVEF: left ventricular ejection fraction.ACE Inhibitor: angiotensin-converting-enzyme inhibitor.

ARB: angiotensin II receptor inhibitor.

#### BNP peptides

(Table **2**) Before depletion, ProBNP_1-108_, NT-proBNP and BNP levels were 316.8+/-265.9 pg/ml, 6,054.0+/-11,539 pg/ml and 684.3+/-1,092 pg/ml respectively.

**Figure 1:**
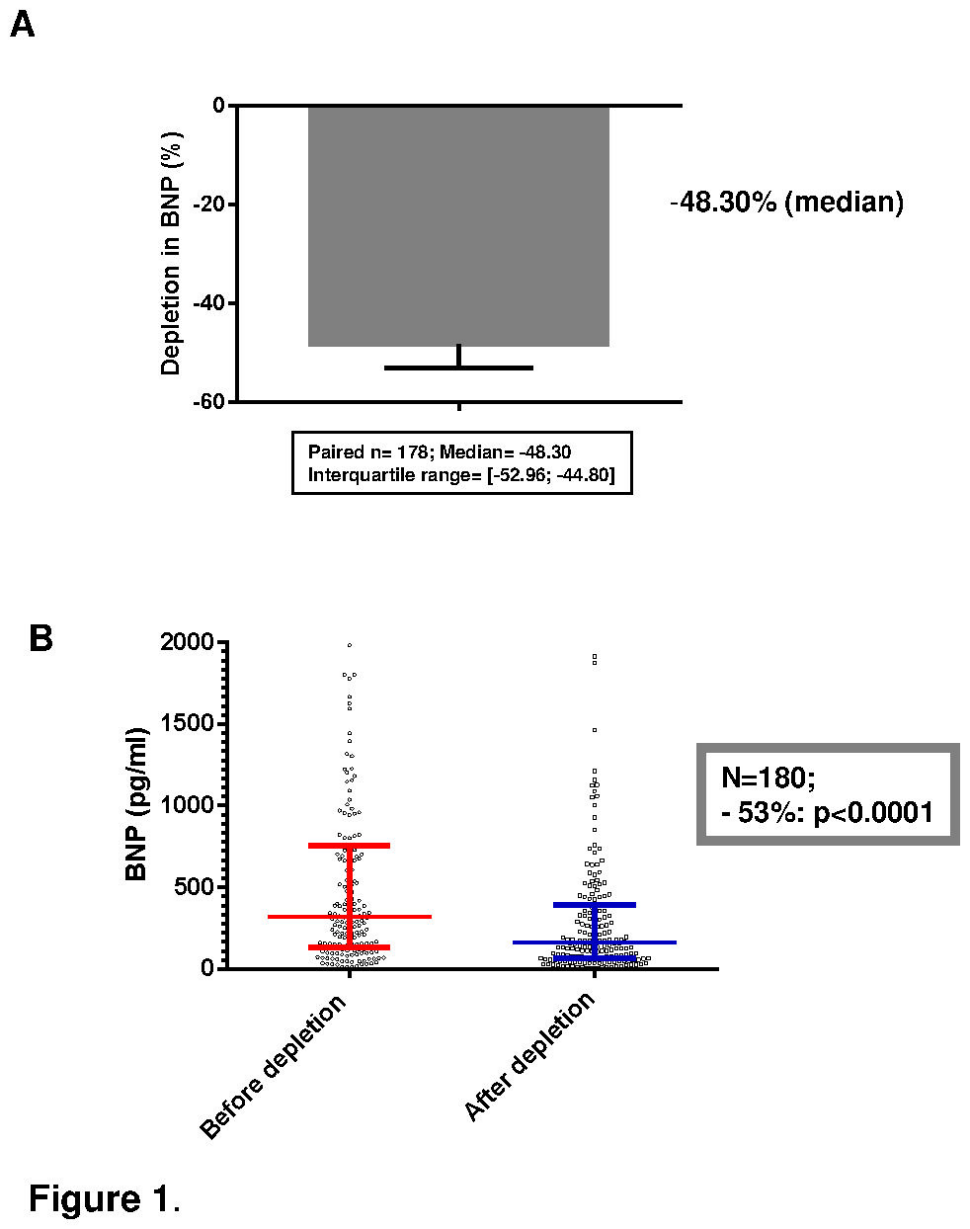
Circulating BNP levels before and after depletion of proBNP_1-108_.^.^ BNP decreased significantly by 53% (when means are compared), p<0.0001. A: Median and interquartile range of reduction for paired values (n=177). B: Statistical distributions for the 180 patients (median and interquartile range, 16 values are out of the axis limits).

**Figure 2:**
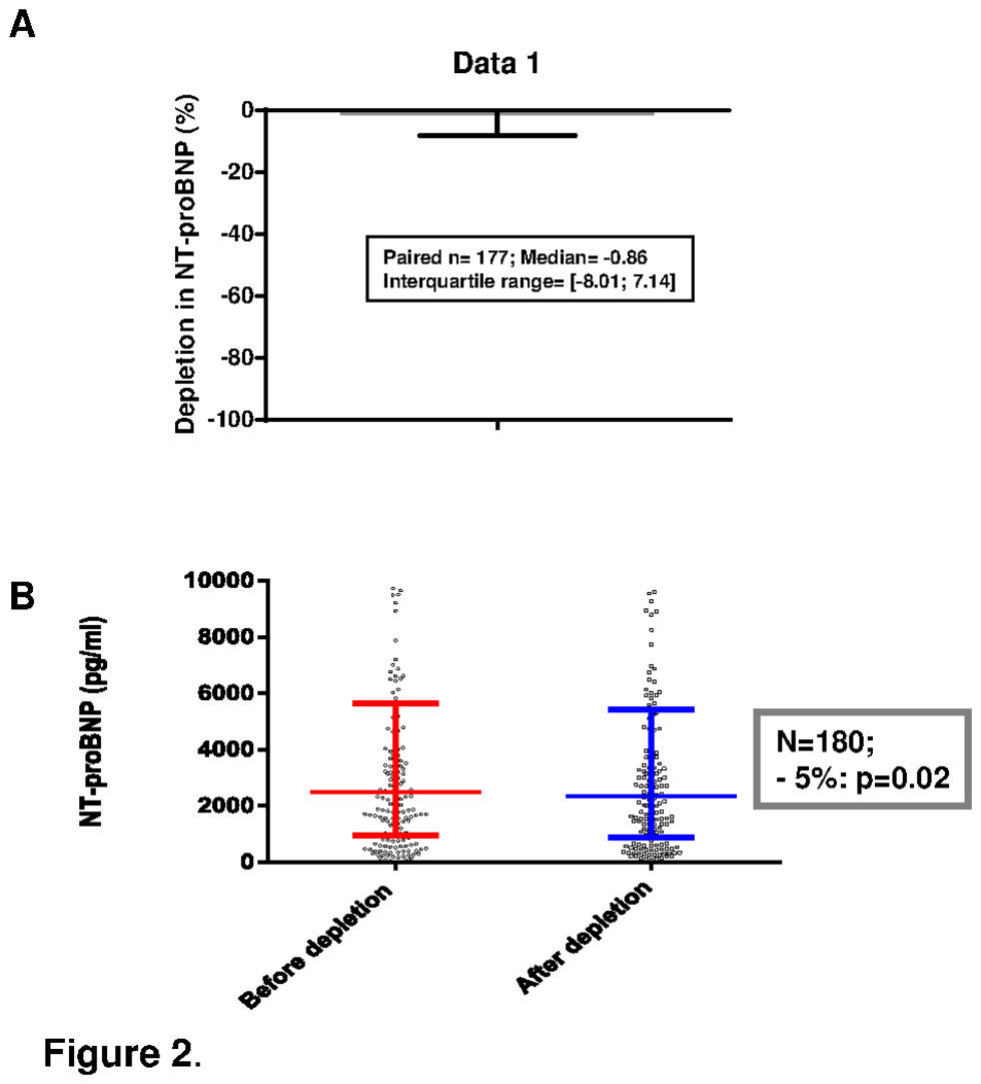
Circulating NT-proBNP levels before and after depletion of proBNP_1-108_. NT-proBNP decreased significantly by 5%, (when means are compared), p=0.02. A: Median and interquartile range of reduction for paired values (n=178). B: Statistical distributions for the 180 patients (median and interquartile range, 48 values are out of the axis limits).

The **technique of depletion** was efficient and led to a total depletion of 96%, p<0.001 (Table **2**). Depletion of proBNP_1-108_ resulted in a deep decrease in BNP (decrease in means was 53% for the 180 samples ; median decrease was 48.3% (interquartile range 44.8%-53.0%) for 178 paired analyses), highly statistically significant (p<0.0001) (Figure 1). It also resulted in a mild decrease in NT-proBNP (decrease in means was 5% for the 180 samples ; median variation was -0.86% (interquartile range -8.0% -+7.1%) for 177 paired analyses), statistically significant (p=0.02) as shown in Figure 2.

**Table 2:** Main biochemical results.

Values*	Before depletion	After depletion	Mean reduction (%)	P value
BNP (pg/ml)	684.3+/-1,092.0	317.9+/-435.3	53%	P<0.0001
NT-proBNP (pg/ml)	6,054.0+/-11,539.0	5,760.0+/-11,510.0	5%	P=0.02
proBNP (pg/ml)	316.8+/-265.9	13.3+/-29.1	96%	P<0.0001

^*^ Plus–minus values are means ± standard deviations.

**Figure 3:**
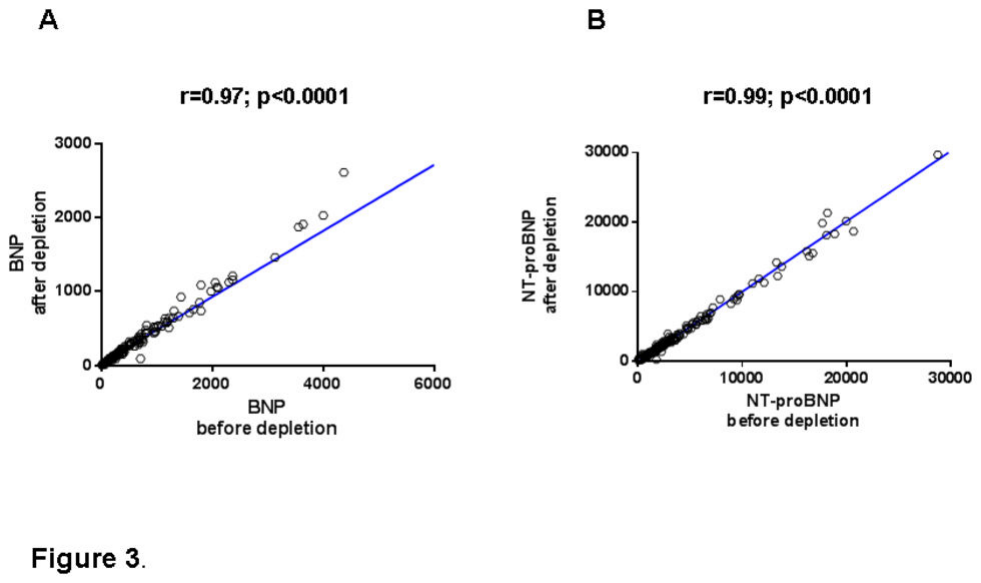
Correlations between BNP and NTproBNP assays before and after depletion of proBNP_1-108_. A: BNP. Correlation between the two assays is very strong, in spite of the deep reduction of the values: n=178, r=0.97; p<0.0001. B: NT-proBNP. Correlation between the two assays is very strong, in spite of the mild reduction of the values N=178, r=0.99; p<0.0001.

**Figure 4:**
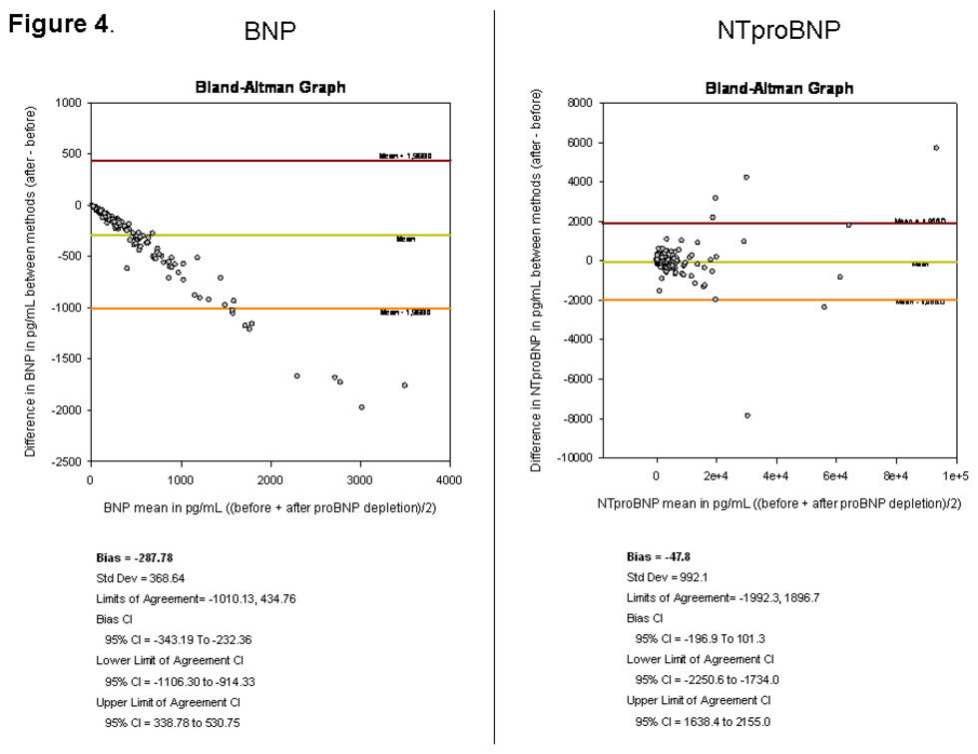
Bland Altman representation of the individual variations before and after depletion, reported to the start level of peptides BNP (A), NT-proBNP (B). A: BNP. The relationship is clearly linear, whatever the start level of BNP before the depletion procedure. B: pro-BNP: the relashionship is not strong.

Correlation coefficients were very good between the two assessments (before and after depletion), as presented on Figure 3, respectively for BNP, r=0.97, p<0.0001; and for NT-proBNP r=0.99, p<0.0001. As shown in the Bland-Altman plot (Figure 4), the magnitude of the BNP difference for each patient increased between both conditions: before and after proBNP depletion. Around 95% of patients have a difference in BNP levels between before and after proBNP depletion (bias=-287.7), whereas the NTproBNP presents no magnitude modification between the two conditions (bias=-47.8).

**Figure 5:**
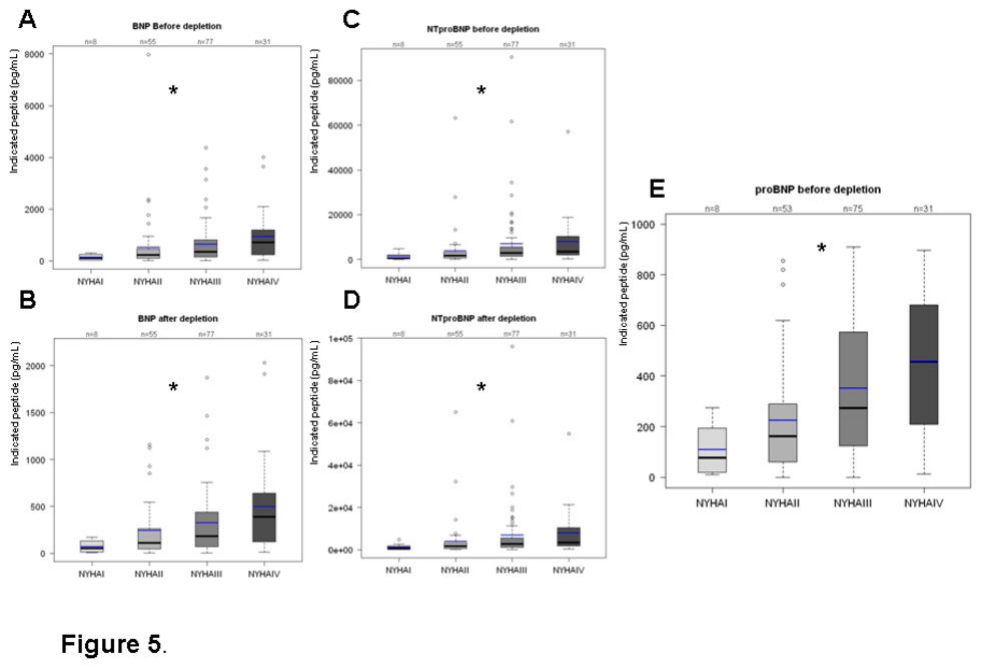
Distribution according to NYHA classes of: BNP before depletion (A), after depletion (B) or NTproBNP before depletion (C), after depletion (D), and proBNP_1-108_ before depletion (E). All of these Natriuretic peptides are strictly correlated with the functional classes, with p<0.001 (specific values after Kruskal-Wallis analyses are indicated).

The three peptides, proBNP, BNP and NT-proBNP, were closely related to NYHA classes. Differential analyses did not reveal significant differences between the **three natriuretic peptides**: all of them are strictly correlated with the functional classes, (data presented on Figure **5**), with regards to discrimination of HF classes, even after depletion. The **mean ratio proBNP_1-108_ to NT-proBNP** was 0.105+/-0.0005, irrespective of the HF stage.

**Figure 6:**
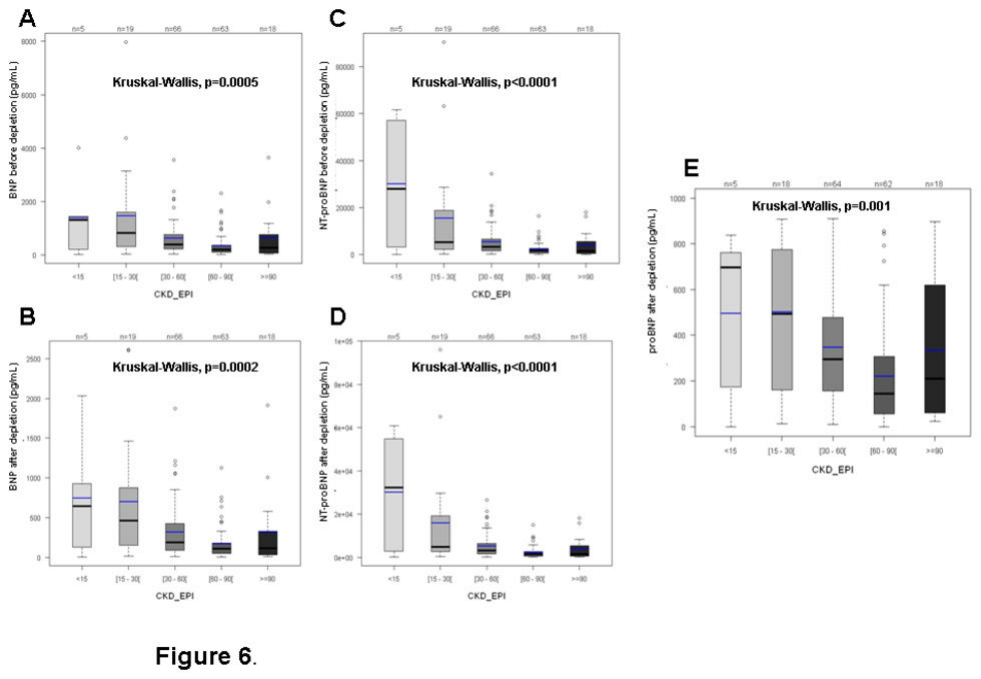
Distribution according to renal function (estimated by CKD-EPI) of: BNP before depletion (A), after depletion (B) or NTproBNP before depletion (C), after depletion (D), and proBNP_1-108_ before depletion (E). All of these Natriuretic peptides are strictly correlated with the renal function, with p<0.001 (specific values after Kruskal -Wallis analyses are indicated).

**Figure 7:**
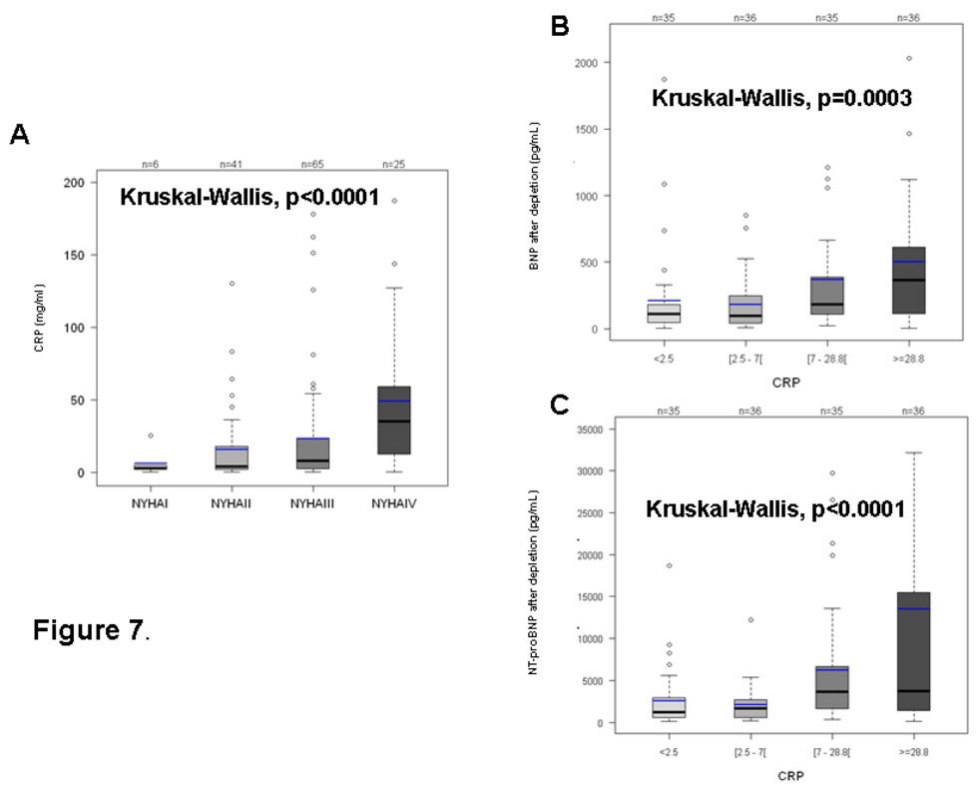
Correlations between CRP, NYHA and BNP or NTproBNP after depletion of proBNP_1-108_. A: CRP is correlated with NYHA. CRP levels increase significantly when the NYHA class becomes higher (P<0.0001). B: BNP after depletion is correlated with CRP levels. BNP levels are considered in quartiles. P=0.0003. C: NT-proBNP after depletion is correlated with CRP levels. BNP levels are considered in quartiles. P<0.0001.

Interestingly, beyond all the three natriuretic peptides that increase significantly following the functional NYHA classes, urea but not creatininemia, and CRP were significantly positively correlated with the NYHA functional classes. They all increase in case of renal impairment and this increase was strictly correlated with the decrease in the renal function (P<0.01 or <0.0001), as represented on Figure **6** (renal function is then estimated by the CKD-EPI, (Chronic Kidney Disease - Epidemiology Collaboration)). CRP not only correlated with NYHA, but also with both BNP and NT-proBNP after depletion, as schematically presented on Figure **7**.

## Discussion

In this study, for the first time, we demonstrated that effective immuno-depletion of the precursor proBNP_1-108_ (up to 96% of the circulating proBNP_1-108_) revealed **a clear cross-reactivity of proBNP in BNP assays** and in a lesser extent of NTproBNP in routine clinical assays in HF management. The BNP levels are overestimated by more than 50% in absence of depletion of proBNP_1-108_. Secondly, we confirm that **intact proBNP_1-108_ is correlated to HF stage and increases in case of renal impairment** (as well as BNP or NT-proBNP). CRP appears to be correlated both to functional classes and natriuretic peptides after depletion deserves to be mentioned, as this correlation is not always so strong in recent studies [11], and as this could explain -at least partly- the progression of the disease [12].

From a clinical point-of-view, the **simultaneous measurement of two peptides** representing on the one hand the biologically inactive intact precursor proBNP_1-108_ and on the other hand the active derived BNP peptide, could allow clinicians to follow better the status of the patient, especially in pathological conditions. For instance, in ambulatory patients with chronic HF, combined assessment of BNP and proBNP provided additional information with regards to risk of adverse clinical outcomes [13]. In these patients, this combined measurement was particularly promising in patients with low levels of BNP, in which the clinicians could be falsely reassured. Clinical evaluation of combinations of highly specific assays for the assessment of BNP peptides are still needed [14] especially in patients with chronic renal failure since the half-life of the 3 peptides and/or their respective renal clearance is poorly understood [15].

Secondly, our findings **rise concerns about the currently available methods for assessing natriuretic peptides**: either to encourage more specific methods, such as the new process presented here, or to redefine cut-off values (especially as regards the “grey zone” even if this was not specifically the point of interest in the present study), taking into account the lack of specificity of the currently used methods. Dries et al showed indeed that simultaneous assessment of unprocessed proBNP_1-108_ in addition to processed BNP_32_ could improve identification of high-risk ambulatory patients with HF [13]. Our method could provide similar information, so that clinical implications deserve to be clarified.

**Surprisingly, the clinical signification** of BNP overestimation remains unclear as the sample pre-treatment technique did not allow us to better stratify the HF patients and did not contribute to a better patient management care. At least two reasons could be proposed.

First, the two different methods for assessment of peptides before or after depletion seem to provide similar clinical information on this HF cohort of patients, as suggested by the very strong correlations between them (at least for BNP).

Secondly, BNP peptides are already powerful tools to stratify HF patients, so that it could be difficult to improve significantly the clinical meaning in a small population or in a real-life population: only class III patient for instance should have been included to get a more specific population with better predefined endpoints, and a long follow-up. This hypothesis is consistent with the study on a specific not commercially available test in a large cohort [16]. Disappointingly, although proBNP_1-108_ was a sensitive (78.8%) and specific (86.1%) biomarker for detecting left ventricular systolic dysfunction, this biomarker was comparable but not better than BNP and was even less informative than NT-proBNP_1-76_ [16], at least in this HF cohort.

In addition, in a recent cohort of 187 Class III-IV HF patients, pro-BNP_1-108_, standard assay BNP and troponin T (cTnT) were investigated in relation to the primary endpoint of death or cardiac transplantation [17]. Both elevated proBNP_1-108_ and BNP were associated with increased clinical events. More importantly, elevated levels of proBNP_1-108_ or BNP identified by serial monitoring similarly predicted events.

The second surprising result of this study is that no significant benefit for immunodepletion could be observed on the NT-proBNP molecule measurement itself (5% decrease for NT-proBNP_1-76_ versus 50% decrease for BNP). Knowing that an equimolar ratio is expected in the proBNP_1-108_ cleavage by furin/corin, one could guess to observe a significant decrease in the NT-proBNP_1-76_ molecule while using the immunodepletion method. To understand this surprising effect, we recognized that O-glycosylated forms of proBNP in heart failure patients were reported, along with uncleaved proBNP [8,18-20]; all reported an overestimation of BNP_1-32_ and NT-proBNP_1-76_ due to the cross-reactivity of antibodies towards the proBNP_1-108_. We therefore hypothesized that the hyperglycosylation of proBNP _1-108_at the 1-76 part [18,19,21] might prevent the accessibility of the anti-hinge antibody during the immunodepletion process and therefore decrease its efficiency. We therefore suggest verifying that by using O-glycosidases, neuraminidase and galactosidase enzymes [21], the immunodepletion will stay the same or will benefit from a better access of the anti-hinge antibody. Furthermore, this limited cross-reactivity of the NT-proBNP assay with the proBNP could result from the impact of glycosylation on the detection of NT-proBNP: NT-proBNP could be underdetected because of the glycosylation of the NT-proBNP [22], which could prevent the antibodies from an accurate recognition.

These findings demonstrate that the “BNP” which is supposed to be measured in various clinical settings is not really the true BNP pool Only few publications have already showed the cross-reactivity among natriuretic peptide assays. Luckenbill et al studied pooled samples of healthy donors with established concentrations and demonstrated that BNP assays crossreact with NT-proBNP or proBNP, and calculated the various differences between 5 commercial BNP and 3 commercial NT-proBNP assays [8]. Hawkridge et al, used an immunoaffinity purification assay to isolate endogenous BNP specifically in the plasma of 4 patients ranged NYHA class IV dedicated to subsequent analysis by nano-liquid chromatography, to evidence the absence of circulating BNP in advanced-stage HF patients and they suggested the existence of altered forms of BNP [23]. Here, in a real-life cohort of 180 patients with HF, we established a similar cross-reactivity corroborating that **the true BNP pool could represent less than 50% of what is measured by a currently used commercial assay**.

Furthermore, it could explain at least partly **discrepancies among commercial kits**, as specificity could vary from one to another, hence a lack of alignment and difficulties to follow patients when different commercial assays are run in different laboratories.

### Limitations

These findings have to be confirmed in a larger population, although this cohort of 180 patients with HF represents a real-life situation. Secondly, it will be of critical importance to test different platforms to demonstrate the general impact of proBNP depletion on BNP levels. Here is provided first technical proof of concept of the impact of proBNP depletion on BNP testing in a pilot study.

In this population, taking into consideration the cross reactivity did not modify the classification of patients. It remains unclear whether considering these true values simply drives all values to 50% lower levels or on the contrary could elicit a profound impact in the classification for specific patients (BNP grey zone, renal impairment, elderly etc). Further larger studies will address this concern.

Finally, it could be suggested that since diagnostic and prognostic performances of BNP and NT-proBNP are roughly similar, if NT-proBNP measurement is not affected by the presence of proBNP, only NT-proBNP and not BNP could be suggested to be used. Nevertheless, as performances are similar, our results could not by themselves support such conclusions. They should on the contrary suggest to develop more specific assays to better understand what is currently assayed (other interactions could obviously be searched as regards NT-proBNP as previously discussed) and propose perhaps more accurate cut-offs.

## Conclusion

Routinely used BNP assays are not specific to BNP molecule but also cross react with the proBNP_1-108_ precursor. By using an immunodepletion method, we were able to really assess the true BNP measurement. Assessing accurately each of the three major natriuretic peptides could be of interest to better characterize patients and to address appropriate therapy especially in case of decompensated heart failure patients. This could open new fields of interest to better understand unclear medical situations and above all to enlighten the grey zone, in order to better stratify patients and to perform a better drug monitoring. Clinical implications of these new methods remain to be investigated.
